# Non-Hepatic Alkaline Phosphatase, hs-CRP and Progression of Vertebral Fracture in Patients with Rheumatoid Arthritis: A Population-Based Longitudinal Study

**DOI:** 10.3390/jcm7110439

**Published:** 2018-11-13

**Authors:** Jih-Chen Yeh, Chang-Chin Wu, Cheuk-Sing Choy, Shu-Wei Chang, Jian-Chiun Liou, Kuo-Shu Chen, Tao-Hsin Tung, Wei-Ning Lin, Chih-Yu Hsieh, Chun-Ta Ho, Ting-Ming Wang, Jia-Feng Chang

**Affiliations:** 1 Department of Dentistry, Far Eastern Memorial Hospital, New Taipei City 220, Taiwan; b202093012@tmu.edu.tw; 2 School of Dentistry, Graduate Institute of Clinical Medicine, College of Medicine, National Taiwan University, Taipei 106, Taiwan; 3 Department of Orthopedics, En Chu Kong Hospital, New Taipei City 237, Taiwan; Dtorth65@yahoo.com.tw; 4 Department of Biomedical Engineering, Yuanpei University of Medical Technology, Hsinchu 300, Taiwan; 5 Department of Orthopaedic Surgery, School of Medicine, National Taiwan University, Taipei 106, Taiwan; 6 Department of Orthopaedic Surgery, National Taiwan University Hospital, Taipei 106, Taiwan; 7 Department of Community Medicine, En Chu Kong Hospital, New Taipei City 237, Taiwan; prof.choy@gmail.com; 8 Department of Nursing, Yuanpei University of Medical Technology, Hsinchu 300, Taiwan; 9 Department of Civil Engineering, National Taiwan University, Taipei 106, Taiwan; changsw@ntu.edu.tw; 10 School of Biomedical Engineering, Taipei Medical University, Taipei 110, Taiwan; jcliou@tmu.edu.tw; 11 Division of Rheumatology, Department of Internal Medicine, En Chu Kong Hospital, New Taipei City 237, Taiwan; jillkaom@yahoo.com.tw; 12 Department of Medical Research and Education, Cheng Hsin General Hospital, Taipei 112, Taiwan; ch2876@gmail.com; 13 Graduate Institution of Biomedical and Pharmaceutical Science, College of Medicine, Fu Jen Catholic University, New Taipei City 242, Taiwan; 081551@mail.fju.edu.tw; 14 Division of Nephrology, Department of Internal Medicine, En Chu Kong Hospital, New Taipei City 237, Taiwan; fish37435@Hotmail.com (C.-Y.H.); earny2002@gmail.com (C.-T.H.); 15 Renal Care Joint Foundation, New Taipei City 220, Taiwan; 16 Program in Nutrition and Food Sciences, College of Human Ecology, Fu Jen Catholic University, New Taipei City 242, Taiwan

**Keywords:** alkaline phosphatase, C-reactive protein, rheumatoid arthritis, vertebral fractures

## Abstract

Background: Interactions and early warning effects of non-hepatic alkaline phosphatase (NHALP) and high-sensitivity C-reactive protein (hs-CRP) on the progression of vertebral fractures (VFs) in patients with rheumatoid arthritis (RA) remain unclear. We aim to explore whether serum concentrations of NHALP and hs-CRP could serve as a promising dual biomarker for prognostic assessment of VF progression. Methods: Unadjusted and adjusted hazard ratios (aHRs) of VF progression were calculated for different categories of serum NHALP and hs-CRP using the Cox regression model in RA patients. The modification effect between serum NHALP and hs-CRP on VF progression was determined using an interaction product term. Results: During 4489 person-years of follow-up, higher NHALP (>125 U/L) and hs-CRP (>3.0 mg/L) were robustly associated with incremental risks of VF progression in RA patients (aHR: 2.2 (95% confidence intervals (CIs): 1.2–3.9) and 2.0 (95% CI: 1.3–3.3) compared to the lowest HR category, respectively). The interaction between NHALP and hs-CRP on VF progression was statistically significant (*p* < 0.05). In the stratified analysis, patients with combined highest NHALP and hs-CRP had the greatest risk of VF progression (aHR: 4.9 (95% CI: 2.5–9.6)) compared to the lowest HR group (NHALP < 90 U/L and hs-CRP < 1 mg/L). Conclusions: In light of underdiagnoses of VFs and misleading diagnosis by single test, NHALP and hs-CRP could serve as compensatory biomarkers to predict subclinical VF progression in RA patients.

## 1. Introduction

Rheumatoid arthritis (RA) has been recognized as the most prevalent inflammatory arthritis worldwide [1]. An incremental increase in risk of vertebral fractures (VFs) in patients with RA has been identified by a myriad of observational studies and meta-analyses [2,3,4,5,6,7,8,9]. Vertebral fracture (VF) is a highly prevalent type of fragility fracture and associated with impaired activities of daily living, reduced pulmonary functions, cardiovascular diseases and increased mortality [10,11,12,13]. Given that less than one-third of VFs are clinically recognized at the event occurrence time and require spine imaging to be diagnosed, the epidemiology of VFs differs from osteoporotic fractures at other skeletal sites [14]. In Taiwan, the average medical cost of fracture per case is more than NT $100,000 (roughly US$3300) for acute care and consumes enormous amounts of national health expenditures [15] In the United States, the medical expenditure for VFs is more than $1 billion annually. Thus identifying useful biomarkers as early warning signs of VFs for risk stratification in RA patients is warranted. Close monitoring of serum concentrations of alkaline phosphatase (ALP) is recommended in the management of chronic kidney disease-mineral and bone disorder (CKD-MBD) [16]. Since ALP isoenzymes are mainly derived from bones and liver, raised serum concentrations of non-hepatic ALP (NHALP) reflect bone pathology [17]. Moreover, C-reactive protein (CRP) is the first-choice biomarker for RA assessment in hospital practice and high sensitivity CRP (hs-CRP) predicts VFs in the elderly population [18,19]. In light of this, we aimed to investigate the increase in serum concentrations of NHALP and hs-CRP reflects the increased risk of VF progression in RA patients. The modification effect between NHALP and hs-CRP on progression of VF was also investigated in this population-based study with relatively long follow-up.

## 2. Methods

### 2.1. Cohort

We enrolled 2069 patients diagnosed with RA (ICD-9 codes: ICD-9-CM codes: 714.0, 714.1, 714.2, 714.30–714.33) through hospital databanks from January 2002 to June 2017. After the enrollment, 1237 patients were excluded due to incomplete laboratory or imaging studies. The majority of excluded patients lacked the data of ALP. Furthermore, 316 patients with evidences of hepatic diseases or elevated gamma-glutamyl transferase were excluded by careful review of medical charts and records. Afterwards, 516 RA patients with complete data were eligible for the final analysis.

### 2.2. Assessment of VFs

All participants underwent at least 2 thoracolumbar imaging studies (baseline reference and follow-up) by radiography or computed tomography (CT). In cases that had both plain radiographs and CT performed, evaluation of VF was conducted on CT images. Follow-up radiography to rule out fractures was conducted when patients complained of lower back pain or experienced a fall. VFs were assessed with Genant’s method using two-dimensional (2D) or three-dimensional (3D) measurements [20]. Image processing techniques in the Vitrea fX software (Vital Images, Minnetonka, MN, USA) were employed to quantify the reduction of 2D area or 3D volume in vertebral bodies. The deformities of vertebral bodies were classified into grade 1–3. A reduction of 20–25% in area or volume was classified into grade 1 fracture, a reduction of 20–40% in area or volume was classified into grade 2 and a reduction of 40% or more was classified into grade 3.

### 2.3. Outcomes and Follow Up

The occurrences of VF in RA patients were examined at baseline. Progression of VF was defined as the first progression of Genant’s Grade or any new-onset osseous disruption of vertebra by imaging studies. To determine the onset timing of VF progression, all spinal images of each RA patient with irregular-interval follow-up were carefully reviewed and analyzed by both orthopedic surgeons and radiologic technicians.

### 2.4. Variables

Bio-demographic data of each patient were collected, including age, sex, hypertension, diabetes mellitus (DM), hyperlipidemia, smoking history and osteoporosis. Osteoporosis was defined as a reduction of 10% or more in area or volume of vertebral bodies at baseline. Baseline serum concentrations of NHALP and hs-CRP were analyzed at first presentation. If the elevated serum concentrations of NHALP and hs-CRP were taken when patients complained of lower back pain, the elevated levels of both biomarkers may actually be due to the fracture. Relevant laboratory variables were also followed up at baseline, such as sodium, potassium, albumin, creatinine (Cr), estimated glomerular filtration rate [21], blood urea nitrogen (BUN), alanine aminotransferase (ALT), fasting glucose, hemoglobin A1c (HbA1c), uric acid, total cholesterol, triglyceride, rheumatoid factor (RF) and erythrocyte sedimentation rate (ESR). The value of eGFR was calculated by the CKD Epidemiology Collaboration (CKD-EPI) equation. We further stratified serum concentrations of hs-CRP into 3 categories (<1.0, 1.0 to 3.0 and >3.0 mg/L) and circulating NHALP into 3 categories (<90, 90 to 125 and >125 U/L).

### 2.5. Statistical Methods

We presented continuous variables as mean ± SD or median with interquartile range and categorical variables as proportions. We calculated Spearman rank correlation coefficients between covariates of interest. We applied Cox regression to model the probability of VF progression. Unadjusted and adjusted hazard ratios (aHRs) of VF progression were calculated for different categories of serum NHALP and hs-CRP in the Cox regression model. The proportional hazards assumption was checked by graphical methods. The modification effect between serum NHALP and hs-CRP on VF progression was determined using an interaction product term. An interaction occurs when the impact of a risk factor on outcome is changed by the value of a third variable, sometimes referred to as effect modification [22,23]. We evaluated if the effect of NHALP on VF progression was modified by hs-CRP through incorporating a multiplicative interaction term in the multivariate model, that is, adding a variable whose value is the product of NHALP and hs-CRP. A significant product term indicates that there is an interaction between NHALP and hs-CRP on the probability of VFs. A *p* value < 0.05 was considered statistically significant. We used PASW Statistics SPSS18 to analyze all bio-clinical data of RA patients. The study had been reviewed and approved by the Research Ethics Review Committee (ECKIRB1061201).

## 3. Results

### 3.1. Characteristics of the Study Population

The final study sample included 516 patients with RA obtaining complete medical records and follow-up. Baseline demographic characteristics and relevant laboratory data of the whole population of study subjects are summarized in Table 1. The mean age was 57.5 ± 12.7 years, approximately 25.2% were male. Prevalence of DM, hypertension, hyperlipidemia and osteoporosis was 22.6%, 31.2%, 16.5% and 23.8%, respectively. The median duration of follow-up was 8.7 years (interquartile range, 5.5–11.9 years). The overall progression rate of VFs was 32.2% during 4489 person-years of follow-up, corresponding to an annual event rate of 3.7%.

The univariate analysis of risk factors associated with progression of VFs among RA patients unveiled that age, male gender, NHALP, hs-CRP and ESR were all significantly associated with progression of VFs (Table 2). Table 3 summarizes the bivariate correlation coefficients between risk factors (NHALP and hs-CRP) and baseline bio-clinical variables in patients with RA. NHALP is positively correlated with age (*r* = 0.20; *p* < 0.05), hs-CRP (*r* = 0.26; *p* < 0.05), osteoporosis (*r* = 0.22; *p* < 0.05), Cr (*r* = 0.16; *p* < 0.05), eGFR (*r* = −0.18; *p* < 0.05) and uric acid (*r* = 0.19; *p* < 0.05), respectively. Moreover, hs-CRP was in turn positively correlated with age (*r* = 0.12; *p* < 0.05), male (*r* = −0.21; *p* < 0.05), osteoporosis (*r* = 0.25; *p* < 0.05), Albumin (*r* = −0.23; *p* < 0.05), ESR (*r* = 0.31; *p* < 0.05) and WBC (*r* = 0.16; *p* < 0.05).

### 3.2. NHALP

**Figure 1:**
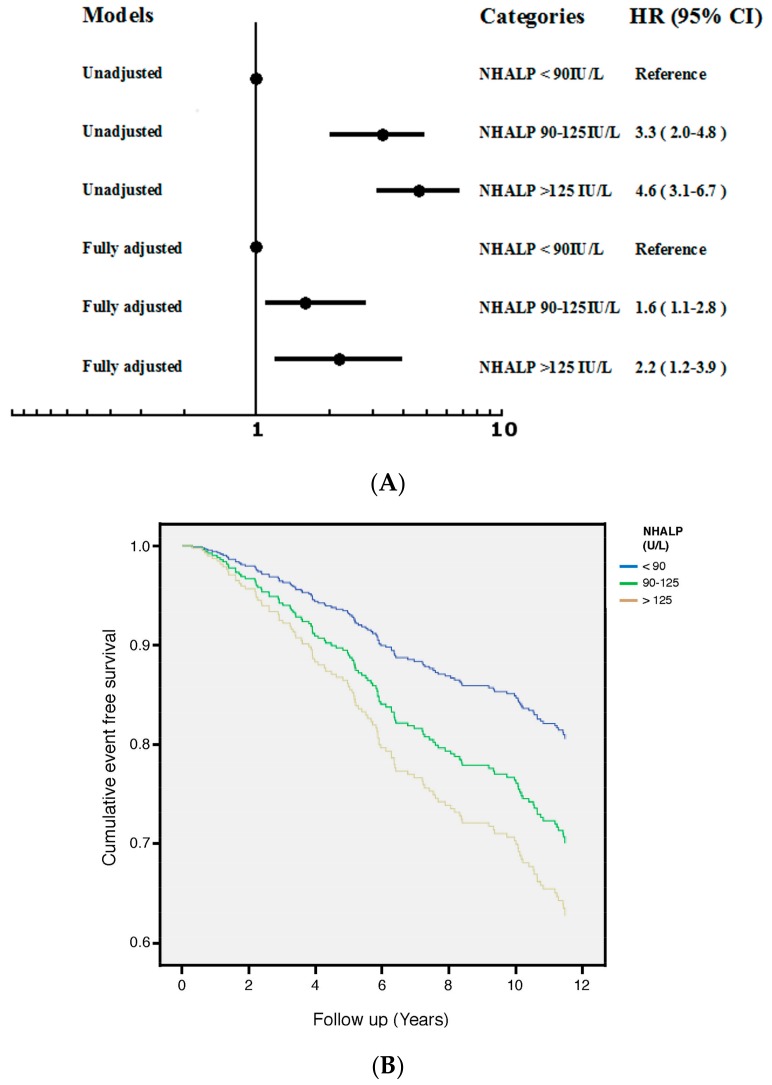
(**A**) Unadjusted and fully adjusted HR of progression of VFs across different categories of NHALP in 516 patients with RA during 4489 person-years of follow-up (Fully adjusted: age, gender, osteoporosis, NHALP, hs-CRP and ESR). The lowest HR group (NHALP < 90 IU/L) served as the reference group. Patients with higher NHALP (>125 IU/L) had the greatest risk of progression of VFs after full adjustments. (**B**) Cumulative survival curves with respect to different categories of NHALP after fully adjusted in the Cox proportional hazards regression model.

Figure 1A illustrated the HR and 95% confidence intervals (CI) for progression of VFs among study patients with different categories of NHALP, considering the lowest HR group (NHALP < 90 IU/L) as reference. Both unadjusted and multivariable-adjusted results demonstrated a robust increase in the HR among patients with higher NHALP after fully adjusted for age, gender, osteoporosis, hs-CRP, NHALP and ESR. Higher levels of NHALP were associated with an increase in the aHR: 2.2 (95% CI: 1.2–3.9) for the category of NHALP > 125 IU/L. Figure 1B showed cumulative survival curves with respect to different categories of NHALP after fully adjusted in the Cox regression model.

### 3.3. hs-CRP

**Figure 2:**
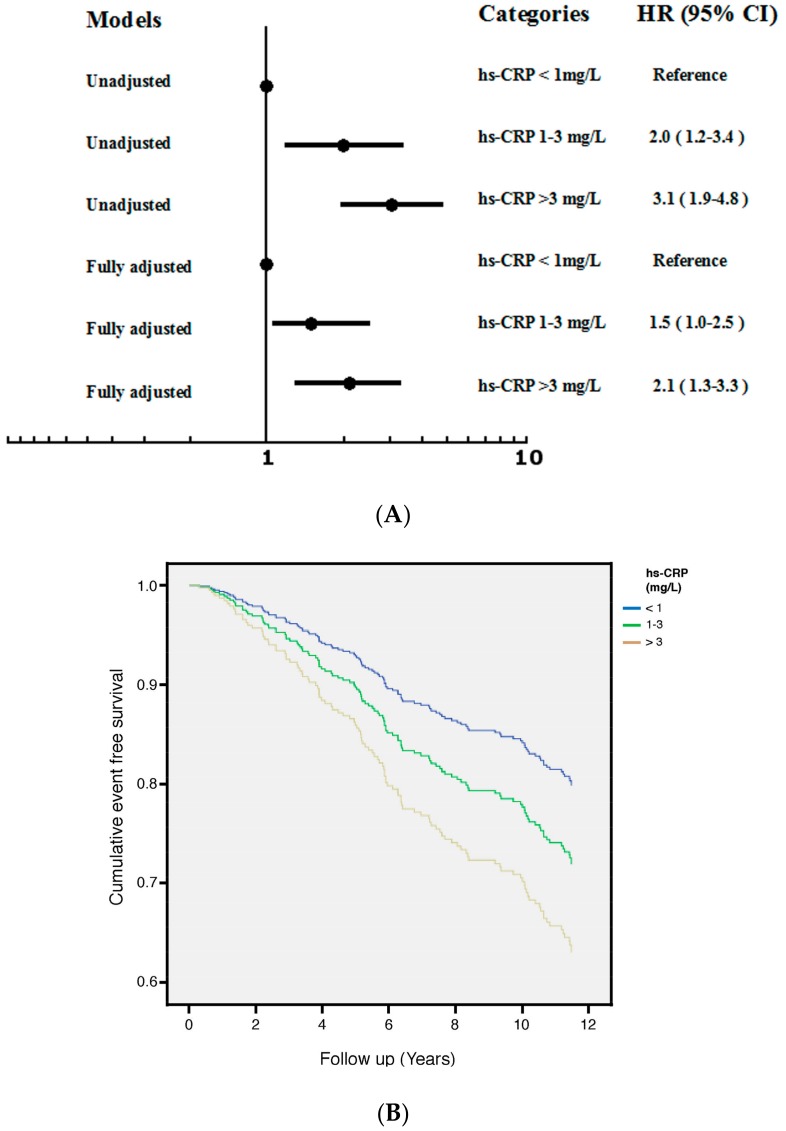
(**A**) Unadjusted and fully adjusted HR of VF progression across different categories of hs-CRP in 516 patients with RA during 4489 person-years of follow-up (Fully adjusted: age, gender, osteoporosis, NHALP, hs-CRP and ESR). The lowest HR group (hs-CRP < 1 mg/L) served as the reference group. Patients with higher hs-CRP (>3 mg/L) had the greatest risk of progression of VFs after full adjustments. (**B**) Cumulative survival curves with respect to different categories of hs-CRP after fully adjusted in the Cox proportional hazards regression model.

Figure 2A illustrated the HR and 95% CI for progression of VFs among study patients with different categories of hs-CRP, considering the lowest HR group (hs-CRP < 1 mg/L) as reference. Both unadjusted and multivariable-adjusted results demonstrated a robust increase in the HR among patients with higher hs-CRP after fully adjusted for age, gender, osteoporosis, hs-CRP, NHALP and ESR. Higher hs-CRP levels were associated with an increase in the aHR: 2.1 (95% CI: 1.3–3.3) for the category of hs-CRP > 3 mg/dL. Figure 2B showed cumulative survival curves with respect to different categories of hs-CRP after fully adjusted in the Cox regression model.

### 3.4. NHALP and hs-CRP

**Figure 3:**
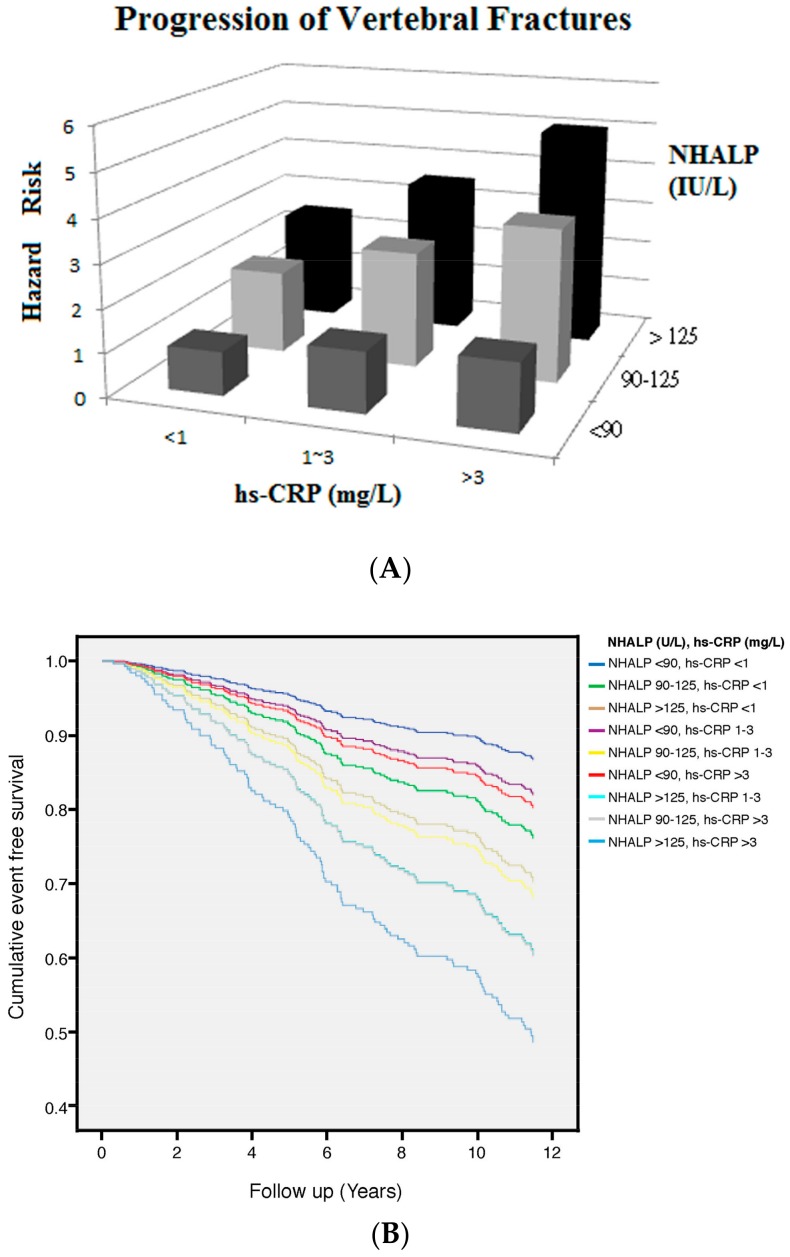
(**A**) Unadjusted and fully adjusted HR of VF progression across different categories of NHALP and hs-CRP in 516 patients with RA during 4489 person-years of follow-up (Fully adjusted: age, gender, osteoporosis, NHALP, hs-CRP and ESR). The reference group (NHALP < 90 IU/L and hs-CRP < 1 mg/L) had the lowest HR. Patients with higher NHALP (>125 IU/L) and higher hs-CRP (>3 mg/L) had the greatest risk of progression of VFs after full adjustments. (**B**) Cumulative survival curves with respect to different categories of NHALP and hs-CRP after fully adjusted in the Cox proportional hazards regression model.

To visually represent the joint effect of two categorical variables on progression of VFs, we utilized three dimensional histograms to illustrate the aHR of progression of VFs across different categories of NHALP and hs-CRP (Figure 3A). Patients with higher NHALP (>125 IU/L) and hs-CRP (>3 mg/L) had the greatest risk of VF progression (aHR: 4.9 (95% CI: 2.5–9.6)) compared to the lowest HR group (NHALP < 90 IU/L and hs-CRP < 1 mg/L). Figure 3B showed cumulative survival curves with respect to different categories of NHALP and hs-CRP after fully adjusted in the Cox regression model. The interaction between NHALP and hs-CRP was statistically significant in the model for effect modification (*p* < 0.05).

## 4. Discussion

Underdiagnosis of subclinical VF events is a worldwide issue [24]. In light of this, we show a brand new idea that the combination of NHALP and hs-CRP is a novel predictor of VF progression among RA patients in a population-based cohort study with relatively long follow-up. In more depth, higher serum concentrations of NHALP and hs-CRP interact to increase the risk of VF progression. Last but not least, NHALP and hs-CRP serve as early warning signs of VFs for risk stratification in RA patients. Several important findings in this work deserve further discussion.

### 4.1. VF Progression and NHALP in RA

RA is an autoimmune disease that causes chronic joint inflammation leading to progressive osteoporosis, high bone turnover and risks of miscellaneous bone fractures [25]. Reducing the medical expenses of bone fractures requires attention to women with early stages of osteopenia [26]. Although measurements of bone mineral density (BMD) are helpful to predict fracture risks, the rates of bone loss are more important for skeletal events with a given BMD [27]. Dynamics of bone turnover and micro-architectural alterations affecting the bone quality can be assessed by bone turnover markers (BTMs) [28]. Given BTMs reflect the rates of bone loss, the decision to treat patients could be based upon fracture risk assessment using BTMs [29]. Since NHALP is the byproduct of active osteoblasts during fracture repair and most widely used BTM [30], monitoring of serum ALP is recommended in the management of CKD-MBD [16]. Our previous research indicates that serum ALP values strongly predict all-cause mortality in 11,912 patients with maintenance hemodialysis [23]. The lowest serum concentrations of ALP have the least possibility of clinical events. To outreach above mechanisms to our current study, the lowest tertile of serum NHALP (<90 IU/L) is defined as our control group. After excluding hepatic diseases, NHALP should be a more specific predictor for high bone turnover events [17]. As expected, higher serum NHALP values were incrementally and independently associated with incremental risks for VF progression before and after multivariable adjustment (Figure 1). Our data demonstrate the pathophysiology between serum NHALP and VF progression involves an increase in bone turnover resulting from an inflammatory milieu of RA. Higher serum NHALP values in RA population reflect pathological bone turnover and thereby contribute to VF progression. To the best of our knowledge, our study is the first one to identify serum concentration of NHALP as a clinically valuable marker to predict events of VF.

### 4.2. VF Progression and hs-CRP in RA

The clinical utility of hs-CRP has been proven as a predictor of various types of bone fractures in elderly men and these fracture events are mainly vertebral [19]. Moreover, the association between hs-CRP and bone fractures was independent of BMD. In their study, the HR of fracture events for the highest tertile of hs-CRP was 1.48 (95% CI, 1.20–1.82), compared with the combined group of the lowest and the medium tertiles. Nonetheless, women with RA have a greater risk of fracture compared to women without RA and sustained VFs at twice the expected frequency [31]. Compared with our study, approximately 75 percent of cases are female. Study participants were divided into tertile groups based on hs-CRP concentrations. Unadjusted and multivariable- adjusted results all demonstrate a robust increase in the HR among patients with higher hs-CRP. Higher hs-CRP levels are associated with an increase in the aHR: 2.1 (95% CI: 1.3–3.3) for the category of hs-CRP > 3 mg/L, compared with the lowest category (Figure 1). Although ESR was closely correlated to hs–CRP and linked to an increased risk of VF progression in univariate analysis (Table 2 and Table 3), the association between ESR and VF progression was not significant after multivariable adjustment. Thus our findings are concordant with previous study showing that joint measurement of serum concentrations of ESR and hs-CRP is unwarranted [18].

### 4.3. Interaction and Joint Effect of NHALP and hs-CRP on VF Progression

Our study demonstrates that age, gender, ESR, hs-CRP and NHALP are all risk factors of VF progression in the univariate analysis. Age, NHALP and hs-CRP are still incrementally and independently associated with increased risk for VF progression after multivariable adjustment for all confounders. Given that both hs-CRP and NHALP were well correlated and associated with VF progression, we assumed that the risk for VF progression would be augmented among RA patients with higher concentrations of hs-CRP and NHALP. Our data unveil that patients with higher hs-CRP (>3 mg/L) and NHALP (>90 IU/L) have the greatest risk of VF progression (Figure 3). The risk for VF progression among RA patients with higher hs-CRP is abated among those with lower NHALP. On the other hand, the risk for VF events among patients with higher NHALP is attenuated among those with lower hs-CRP levels. The interaction between hs-CRP and NHALP on VF risks was statistically significant (*p* < 0.05 for the interaction term). NHALP- and hs-CRP-driven pathways do overlap in VF progression among RA patients and the strong modification effect substantiates the fact that they share common pathogenesis. Current results suggested that NHALP should be routinely measured with hs-CRP and monitored together in clinical practice. NHALP and hs-CRP may serve as a complementary surrogate marker to assess fracture risks in RA population.

### 4.4. Limitation

Our study has several limitations. To begin with, the cross-sectional laboratory values may not reflect substantial intra-individual variability over time. Next, continuous data of BMD are incomplete because our study per se is a retrospective cohort. Furthermore, another limitation is the absence of precise correlations between serum biomarkers, RA disease activity score and therapeutic agents, for example, glucocorticoids. Practice variations in the monitoring disease activity and therapeutic strategies of RA lead to channeling biases. Besides, a high percentage (59.8%) of RA patients was excluded due to incomplete laboratory data that result in a potential biases. Last but not least, the follow-up intervals of spinal images in RA patients are irregular that diagnoses of subclinical VF progression may not be in time.

## 5. Conclusions

VF, a highly prevalent type of fragility fracture in RA patients, is underdiagnosed at the event occurrence time. Both serum concentrations of NHALP and hs-CRP are crucial surrogate markers for high turnover bone diseases, yet there are certain degrees of limitations that mislead to diagnosis of subclinical VFs by single test. Our study demonstrates that combined serum concentrations of NHALP and hs-CRP could serve as a compensatory surrogate marker of VF progression, suggesting NHALP and hs-CRP share the common pathogenic pathway in RA (Supplementary Figure S1). In clinical routine practice, joint measurements of serum concentrations of NHALP and hs-CRP are warranted in RA patients. To avoid underdiagnoses of skeletal events, clinicians should arrange further imaging study for RA patients with an interval increase in both biomarkers.

## Figures and Tables

**Table 1:** Baseline bio-demographic characteristics and relevant laboratory data of the whole study cohort in 516 patients with rheumatoid arthritis.

Variable	Mean ± Standard Deviation or Median (Interquartile Range)
Age (years)	57.5 ± 12.7
Male (*n*, %)	130 (25.2%)
Time (years) 4322 (45.4)	8.7 (5.5–11.9)
Osteoporosis (*n*, %)	123 (23.8%)
Hypertension (*n*, %)	161 (31.2%)
Diabetes (*n*, %)	119 (22.6%)
Hyperlipidemia (*n*, %)	85 (16.5%)
Smoking (*n*, %)	70 (13.6%)
Albumin (g/dL)	4.1 ± 0.5
ALT (IU/L)	26.3 ± 5.7.
BUN (mg/dL)	17.1 ± 13.4
Cr (mg/dL)	0.9 ± 0.8
eGFR (mL/min)	65.7 ± 41.0
NHALP (IU/L)	101.9 ± 70.3
Potassium (mmol/L)	4.0 ± 0.6
Sodium (mmol/L)	138.6 ± 4.2
HbA1c (% of Hb)	6.9 ± 2.3
Fasting glucose (mg/dL)	122.1 ± 76.5
Total cholesterol (mg/dL)	196 (168–220)
Triglyceride (mg/dL)	97 (66–140)
hs-CRP (mg/L)	1.5 (0.8–3.5)
Hb (g/dL)	12.8 ± 1.9
WBC (10^3^/L)	8.2 ± 3.3
ESR (mm/h)	43.7 ± 30.6
RF (IU/mL)	70.5 (18.3–236.0)

Abbreviations: ALT, alanine aminotransferase. BUN, blood urea nitrogen. Cr, creatinine. hs-CRP, high sensitivity C-reactive protein. eGFR, estimated glomerular filtration rate. ESR, erythrocyte sedimentation rate. Hb, hemoglobin. NHALP, non-hepatic alkaline phosphatase. RF, rheumatoid factor. WBC, white blood cells. HbA1c, hemoglobin A1c.

**Table 2:** The correlation coefficients between serum hs-CRP, non hepatic alkaline phosphatase (NHALP) and various bio-clinical variables.

Variable	hs-CRP	NHALP
Age	0.12 *	0.20 *
Male	−0.21 *	−0.02
Smoking history	0.13	−0.05
Hypertension	0.18	0.13
Hyperlipidemia	0.15	0.10
Osteoporosis	0.25 *	0.22 *
hs-CRP	1	0.26 *
NHALP	0.26 *	1
Albumin	−0.23 *	−0.21
BUN	−0.05	0.08
Cr	0.09	0.16 *
eGFR	−0.11	−0.18 *
RF	0.06	0.05
ESR	0.31 *	0.11
Potassium	0.08	0.09
Sodium	−0.01	−0.15
Uric acid	0.19	0.19 *
ALT	0.05	0.17
Total cholesterol	0.14	0.21
Triglyceride	0.19	0.23
Hb	0.12	0.08
WBC	0.16 *	0.13

Note: * *p* < 0.05. ALT, alanine aminotransferase. BUN, blood urea nitrogen. Cr, creatinine. hs-CRP, high sensitivity C-reactive protein. eGFR, estimated glomerular filtration rate. ESR, erythrocyte sedimentation rate. Hb, hemoglobin. NHALP, non-hepatic alkaline phosphatase. RF, rheumatoid factor. WBC, white blood cells.

**Table 3:** Univariate analysis of risk factors associated with progression of vertebral fracture among 516 patients with rheumatoid arthritis.

Variable	Univariate HR for Vertebral Fracture (95% CI)
Age (years)	1.016 (1.009–1.023)
Male	0.705 (0.562–0.976)
Smoke history	0.952 (0.872–1.031)
Hypertension	1.651 (0.933–2.369)
Osteoporosis	2.026 (1.538–3.689)
BUN (mg/dL)	1.004 (0.989–1.020)
Cr (mg/dL)	1.008 (1.000–1.017)
eGFR (mL/min/1.73m^2^)	0.992 (0.980–1.003)
Sodium (mmol/L)	0.931 (0.866–1.001)
Potassium (mmol/L)	1.105 (0.698–1.748)
ALT (IU/L)	1.006 (0.998–1.012)
NHALP (IU/L)	1.005 (1.003–1.007)
HbA1c (%)	1.022 (0.894–1.168)
Fasting glucose (mg/dL)	1.000 (0.996–1.004)
Albumin (g/dL)	0.748 (0.501–1.035)
Total cholesterol (mg/dL)	0.998 (0.991–1.004)
Triglyceride (mg/dL)	1.001 (0.999–1.002)
Uric acid (mg/dL)	1.115 (0.990–1.256)
RF (IU/mL)	1.000 (0.999–1.001)
ESR (mm/h)	1.012 (1.005–1.019)
hs-CRP (mg/L)	1.126 (1.050–1.220)
WBC count (1000 cells/uL)	1.117 (0.997–1.238)
Hb (g/dL)	0.993 (0.844–1.169)

Abbreviations: HR, hazard ratio. CI, confidence intervals. ALT, alanine aminotransferase. BUN, blood urea nitrogen. Cr, creatinine. hs-CRP, high sensitivity C-reactive protein. eGFR, estimated glomerular filtration rate. ESR, erythrocyte sedimentation rate. Hb, hemoglobin. NHALP, non-hepatic alkaline phosphatase. RF, rheumatoid factor. WBC, white blood cells. HbA1c, hemoglobin A1c.

## Data Availability

The numeric data used to support the findings of this study are available from the corresponding author upon request. Corresponding author Jia-Feng Chang email: cjf6699@gmail.com.

## Supplementary Materials

The following are available online at http://www.mdpi.com/2077-0383/7/11/439/s1, Figure S1: Schematic diagram of inflammatory mechanisms of vertebral fractures in patients with rheumatoid arthritis.
