# Supplementary material for: Non-Hepatic Alkaline Phosphatase, hs-CRP and Progression of Vertebral Fracture in Patients with Rheumatoid Arthritis: A Population-Based Longitudinal Study
Source: J Clin Med. 2018 Nov 13;7(11):439. doi: 10.3390/jcm7110439 (PMC6262279; doi:10.3390/jcm7110439)
Supplement: Supplementary file 1 [file jcm-07-00439-s001.pdf]

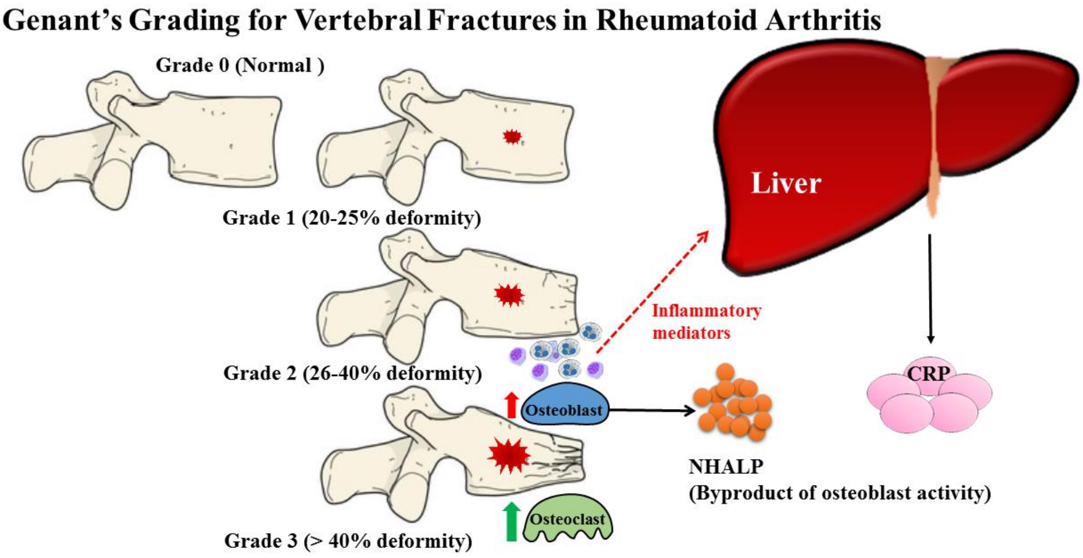

Figure S1. Schematic diagram of inflammatory mechanisms of vertebral fractures in patients with rheumatoid arthritis. Cytokines and responding cells involved in the pathogenesis of rheumatoid arthritis (RA) are more complex than illustrated. Inflammatory mediators stimulate liver to synthesize high sensitivity C-reactive protein (hs-CRP), and thereby increase serum concentration of hs-CRP. The sustained inflammation results in synovitis and progressive damage to cartilage and bone, leading to increased risks of vertebral fractures (VF) over time. Non-hepatic alkaline phosphatase (NHALP) as the byproduct of active osteoblasts is increased during fracture repair. Collectively, hs-CRP and NHALP share the common pathogenic pathway to increase risks of VF in RA patients. Raised serum concentrations of hs-CRP and NHALP could serve as compensatory biomarkers to predict subclinical VF events in bone cases.
